# *Plasmodium falciparum* putative Hsp90 functionally complements yeast Hsp90 and is inhibited in an isoform-specific manner

**DOI:** 10.3389/fpara.2026.1944050

**Published:** 2026-10-12

**Authors:** Shradha Ravindra Khamitkar, Monalisa Bag, Vaibhav R. Kini, Mansi Singh, Badal Kumar Sethi, Sunanda Bhattacharyya

**Affiliations:** Department of Biotechnology and Bioinformatics, School of Life Sciences, University of Hyderabad, Hyderabad, Telangana, India

**Keywords:** CCT018159 IC50 in *Plasmodium*, *Plasmodium falciparum* Hsp90α, *Plasmodium falciparum* Hsp90β, *Plasmodium falciparum* putative Hsp90, yeast complementation

## Abstract

*Plasmodium falciparum* Hsp90α has been proven to be an antimalarial target. However, high sequence conservation between PfHsp90α and HsHsp90α is an obstacle to developing selective inhibitors against the parasite chaperone. The parasite harbours an uncharacterised paralogue of Hsp90α, namely putative PfHsp90 (PF14_0417), which has low sequence conservation with PfHsp90α. A piggyBac transposon insertional mutagenesis study has indicated that putative PfHsp90 is essential for parasite survival. However, its poor sequence conservation with PfHsp90α raises concerns about its functionality. Here, we show that the putative isoform can complement a temperature-sensitive *Schsp82* mutant, confirming its Hsp90-like activity. To identify selective inhibitors against this isoform, we developed a yeast-based assay system using isogenic *Schsp82* temperature-sensitive *Δpdr5* yeast strains, each harbouring *Plasmodium* Hsp90 isoforms. Utilising *bona fide* Hsp90 inhibitors, we measured their *in silico* affinities towards the available structure of putative PfHsp90 and categorised them in increasing order of binding affinities towards the putative isoform. The top-scoring inhibitors were tested within surrogate yeast systems. Our study indicates that only one compound, CCT018159, manifests selective inhibition of the putative PfHsp90 but remains ineffective against PfHsp90α. When tested in the asexual growth of the drug-sensitive and drug-resistant parasites, CCT018159 showed antiparasitic activity in the micromolar range. Together, our study underlines the importance of putative PfHsp90 in parasite survival and merits additional research.

## 1 Introduction

Malaria is a life-threatening disease caused by the *Plasmodium* parasite. According to the World Health Organisation (WHO), there are 282 million reported cases of malaria in 2024, with an estimated 610,000 deaths occurring worldwide (World Malaria Report, 2025). Unfortunately, there was an increase of 9 million cases compared to 2023 due to the development of artemisinin-resistant parasites. There is increasing evidence across the world that parasites are developing resistance to the most potent drugs, artemisinins, chloroquine, and sulphadoxine (Abebe and Woldesenbet, 2025). Current research focuses on the identification of novel targets essential for parasite survival.

The parasite proteome encounters significant temperature alterations within the vertebrate and mosquito hosts, which requires regulation in protein synthesis and stabilisation. The presence of asparagine (Asn) repeat sequences in the *Plasmodium* proteome enhances the propensity for protein aggregation during heat shock conditions. The parasite deploys arrays of molecular chaperones to inhibit protein unfolding and aggregation. The *Plasmodium* Hsp110–Hsp70–Hsp40 chaperone system prevents the aggregation of Asn-rich proteins of malaria parasites (Muralidharan et al., 2012). Heat shock protein 90 plays a pivotal role in protein quality control and has taken a central stage in the development of antimalarials in *Plasmodium*. PfHsp90 chaperone acts as a master regulator of proteostasis by chaperoning the 26S proteasome (Mansfield et al., 2024). It was observed that geldanamycin causes inhibition of the ATPase activity of PfHsp90α (Pallavi et al., 2010), and its derivative 17-AAG can attenuate the growth of *Plasmodium berghei*, thereby allowing prolonged survival of parasite-infected mice (Banumathy et al., 2003). Despite the promising indices from geldanamycin, its poor solubility and high hepatotoxicity became the major limitations in clinical testing as an antimalarial drug. Its derivative tanespimycin (17-AAG or 17-*N*-allylamino-17-demethoxygeldanamycin) exhibited increased potency and less toxicity than geldanamycin (Hoter et al., 2018). However, the repurposing of 17-AAG for malaria was not very effective, as it lacked selectivity for parasite Hsp90s.

The structure of the N-terminal ATP-binding domain of PfHsp90α (PDB: 3K60) reveals a highly similar structure to that of HsHsp90α, and it binds to geldanamycin in an identical manner to that of the human orthologue (Corbett and Berger, 2010). The structural insight led to the identification of novel Hsp90 inhibitors that were shown to be effective at inhibiting *P. falciparum* growth (Shahinas et al., 2013; Zininga and Shonhai, 2019; Posfai et al., 2018; Wang et al., 2014, 2016). High-throughput screening (HTS) of inhibitors was employed to generate more parasite-selective synthetic inhibitors for Hsp90α. Acrisorcin, Harmine, and 2-amino-3-phosphopropionic acid (APPA) were shown to be promising inhibitors of the parasite Hsp90α (Shahinas et al., 2012, 2013, Perković et al., 2020; Marinović et al., 2020). Nonetheless, the compounds exhibited reduced effectiveness against chloroquine-resistant parasite strains. This suggests that although promising, these inhibitors may have other properties that can be circumvented by the parasites, in the same way as chloroquine. More recently, small molecule inhibitors, namely BX-2819, XL888, and Tropane 1, have been identified as potent PfHsp90 inhibitors (Mansfield et al., 2024), of which Tropane 1 displayed about 9.6-fold increased selectivity for PfHsp90 over HsHsp90.

In *Saccharomyces cerevisiae* as well as in humans, there are 96.9% and 85.8% sequence identities between the constitutive and inducible isoforms of Hsp90, respectively. There are functional differences between Hsp90α and Hsp90β in eukaryotes. In mammals, conditional loss of Hsp90α disrupts spermatogenesis, highlighting its reproductive significance, whilst constitutively expressed Hsp90β is critical for embryogenesis (Kajiwara et al., 2012; Chang et al., 2023). In *Arabidopsis thaliana*, α-type isoforms mediate stress responses and hormone signalling, whilst β-type isoforms maintain protein homeostasis and organelle function essential for growth (Cha et al., 2013). The sole cytosolic Hsp90 homologue of *Caenorhabditis elegans*, DAF-21, reflects β-type Hsp90 functionality, fulfilling essential chaperone functions (Chadli et al., 2008).

*Plasmodium* harbours an uncharacterised paralogue of Hsp90, namely, putative Hsp90 (PF3D7_1443900/PF14_0417). A *piggyBac* transposon insertional mutagenesis study indicated that this isoform is essential for parasite survival (Zhang et al., 2018). However, its exact function within the parasite remains elusive. The putative Hsp90 isoform and PfHsp90α are structurally distinct and share only 27.6% sequence identity. The crystal structure of the amino-terminal domain of the putative Hsp90 isoform (PDB: 3IED) shows that the protein contains a unique ATP-binding cleft as compared with PfHsp90α. Furthermore, this isoform is distinct from eukaryotic Hsp90 (28% sequence identity with human Hsp90α/β) as it lacks the charged linker region and the MEEVD motif at the C-terminal end of the protein, which are essential for association with certain cochaperones (Dutta et al., 2022; Gahlawat et al., 2025). Hence, characterisation of this isoform may allow identification of highly selective compounds with minimal cross-reactivity with the host protein.

In the present study, using yeast as a surrogate model system, we have established for the first time that putative Hsp90 has an Hsp90-compatible chaperone activity. Furthermore, using *in silico* approaches, we measured the binding affinities of *bona fide* Hsp90 inhibitors towards the putative isoform of PfHsp90 and predicted promising inhibitors for this isoform. Using a yeast-based assay system, we found that one of the predicted inhibitors showed selectivity for putative PfHsp90. Furthermore, we investigated its inhibitory effects on the asexual developmental growth of *Plasmodium falciparum*.

## 2 Materials and methods

### 2.1 Plasmids

*PfHsp90β* was amplified using trophozoite stage-specific cDNA as a template and primer pairs OSB675 (5′-GGATCCATGCAAAATGTTTATGTTGGGAAC-3′) and OSB676 (5′-GTCGACCTATAATAGTGTTTTATCAGAACC-3′), which had *BamHI* and *SalI* sites incorporated in the forward and reverse primers, respectively. The insert was cloned into the *pHCA* vector (Laskar et al., 2011) and confirmed by DNA sequencing (Eurofins). Subsequently, *PfHsp90α*, *PfHsp90β*, and *ScHsp82* were individually subcloned into the *TRP* marker-containing yeast vector *pTA* (Fangaria et al., 2022) and confirmed by restriction digestion.

### 2.2 Yeast strains

The yeast strains used in this study are listed in **Supplementary Table S1**. For the plasmid shuffling assay, we transformed the *pHCA-PfHsp90β* plasmid into the yeast strain *P82a* (Nathan and Lindquist, 1995) harbouring the *ScHsp82* plasmid with a *TRP* marker (Suhane et al., 2015). For the genetic complementation assay using a temperature-sensitive yeast strain, we transformed the empty *pHCA*, *pHCA-ScHsp82*, and *pHCA-PfHsp90β* plasmids individually into the temperature-sensitive mutant strain *iGD170hsp82* to generate *MBY1*, *MBY2*, and *MBY3* strains, respectively. For determining the sensitivity of inhibitors in a yeast-based assay system, we used the *DP533* strain, which was a generous gift from Professor Didier Picard. In that strain, endogenous *Hsp82*, *Hsc82*, and *PDR5* were knocked out, and cells were maintained with a 2-µm yeast plasmid (*URA3* marker) expressing *HSC82*. We transformed an integrating plasmid, *piHGpd/G170D* (Addgene), that harbours the *hsp82* temperature-sensitive mutant (G170D) driven by the GPD promoter. We grew the generated strain in the presence of 5-fluoroorotic acid (5-FOA) to ensure the loss of *the HSC82-*expressing *URA* plasmid. The newly generated strain (SLY89) supports cell growth like wild-type at the permissive temperature (25°C) due to the presence of the *G170Dhsp82* mutant. We generated three isogenic yeast strains *MBY4*, *MBY5*, and *MBY6* by transformation with 2-µm yeast expression vectors, *pTA-ScHsp82*, *pTA-PfHsp90α*, and *pTA-PfHsp90β*, respectively, into the SLY89 yeast strain.

### 2.3 Growth assay

Three isogenic strains *MBY1*, *MBY2*, and *MBY3* were tested for a genetic complementation study. Cultures were grown overnight in YPD medium at the permissive temperature (25 °C). The next day, secondary cultures were grown to an OD_600_ of 0.2 at 25°C and divided into two parts; one set was allowed to grow at the permissive temperature 25°C and the other set was exposed to the restrictive temperature 37°C for either 4 or 8 h. Subsequently, OD_600_ was measured, all cultures were normalised, and about 1,000 cells were spread on YPD agar plates and incubated for 24 h at 25 °C. Subsequently, the colonies obtained from those grown at 25 °C versus those grown at 37 °C were counted. The percentage survival was calculated for each strain using the formula:


$$\begin{array}{c} \%\text{ Survival }=\;(\left[\text{Number of colonies obtained at }37\;{}^{\circ}\text{C}\right]/\\ \left[\text{Number of colonies obtained at }25{}^{\circ}\text{C}\right])\;\times \;100. \end{array}$$

For drug assays, the above protocol was followed with minor modifications. Typically, freshly cultured *MBY4*, *MBY5*, and *MBY6* strains were grown at 25 °C overnight and subsequently used to inoculate the secondary culture. The secondary cultures with an OD_600 nm_ of 0.2 were divided into two parts, with one incubated at 37 °C for 4 h and the other subjected to different doses of six individual drugs, namely: 2 µM radicicol (Sigma, St. Louis, MO, USA), 20 µM 17-AAG (Sigma), 2 µM NVP-AUY922 (MedChemExpress), 50 µM CCT018159 (MedChemExpress), 6 µM ganetespib (MedChemExpress), and 20 µM MPC-3100 (MedChemExpress) for 4 h at 37 °C. Subsequently, 1,000 cells from each of the untreated and inhibitor-treated cultures were spread on YPD agar plates and incubated at 25°C for 24 h. The percentage survival was calculated as per the formula:


$$\begin{array}{c} \%\text{ Survival }=\;(\left[\text{Number of colonies obtained with added inhibitor}\right]/\\ \left[\text{Number of colonies obtained without inhibitor}\right])\;\times \;100. \end{array}$$

### 2.4 *Plasmodium falciparum* culture

In this study, we employed two reference strains of *Plasmodium falciparum* for *in vitro* culture: the drug-sensitive 3D7 strain and the multidrug-resistant Dd2 strain. Parasite growth was maintained in human erythrocytes at 5% haematocrit in RPMI 1640 medium (Himedia) supplemented with 0.005% (v/v) hypoxanthine (Sigma) and 0.5% (w/v) Albumax (Thermo Fisher Scientific), with cultures incubated at 37 °C using the candle jar method. Synchronisation at the ring stage was achieved by treatment with 5% sorbitol (Sigma, St. Louis, MO, USA) (Bansod et al., 2020). Parasitemia was assessed by microscopic examination of Giemsa-stained slides by counting infected erythrocytes. Following synchronisation, parasites were harvested at the ring (18–20 hpi), trophozoite (30–32 hpi), and schizont (44–45 hpi) stages.

### 2.5 RNA isolation and reverse transcription-polymerase chain reaction analysis

Synchronised *P. falciparum* 3D7 parasites were collected at the ring, trophozoite, and schizont stages, and total RNA was extracted using the TRIzol method according to our previously established protocol (Singh et al., 2023). For *Saccharomyces cerevisiae*, *MBY4*, *MBY5*, and *MBY6* strains were evaluated for expression analysis. A total OD_600 nm_ of 10 for each cell strain was harvested, and RNA was extracted using the acid-phenol method (Laskar et al., 2011). To eliminate residual DNA from both parasite and yeast preparations, 5 µg of RNA samples were treated with DNase I (Fermentas), and the absence of genomic DNA contamination was confirmed by PCR reactions performed without reverse transcriptase. Subsequently, cDNA synthesis for both organisms was performed using 1 µg of DNase-treated RNA with reverse transcriptase (Qiagen), and gene expression was analysed by semiquantitative RT-PCR amplification with specific primers. The expression of *PfHsp90β* was measured using the primers OSB676 (5′-GACGTCGACCTATAATAGTGTTTTATCAGAACC-3′) and OSB686 (5′-TACATCTGGTTCGGAAATGCC-3′), which amplified a 317-base pair fragment of the transcript. We utilised OSB94 (5′-CTGTAACACATAATAGATCCGAC-3′) and OSB95 (5′-TTAACCATCGTTATCATCATTATTTC-3′) to amplify 300-bp gene-specific regions of *P. falciparum* aspartate-rich protein (*PfARP*), as a loading control, which is constitutively expressed during the asexual stage of the parasite (Dobson et al., 2003). The expression of *ScHSP82* was assessed with the aid of primers OSB687 (5′-TCCTCCTACATGTCTTCCAAG-3′) and OSB22 (5′-CGGGTCGACCTAATCTACCTCTTCCATTTCGG-3′), which amplified a 291-bp transcript. The primer pair OSB14 (5′-TTAGAAACACTTGTGGTGAACG-3′) and OSB16 (5′-TGACCAAACTACTTACAACTCC-3′) was used to amplify a 307-bp fragment from the 3′ end of the *Actin* transcript, which served as a loading control for normalisation of target gene transcript levels in yeast. We quantified the band intensities using ImageJ software and normalised target gene expression with respect to the expression of *ARP*.

### 2.6 IC_50_ determination of CCT018159 in drug-sensitive and drug-resistant parasites

To determine the half-maximal inhibitory concentration (IC_50_) of CCT018159, tightly synchronised ring-stage as well as late trophozoite-stage specific *Plasmodium falciparum* 3D7 (drug-sensitive) and Dd2 (multidrug-resistant) parasites were treated with different concentrations of the inhibitor (0.1 nM, 1 nM, 10 nM, 100 nM, 1 µM, 10 µM, and 100 µM) for one complete 48-h intraerythrocytic developmental cycle at 37 °C. Parasite survival was assessed by preparing blood smears from all treatment groups, followed by methanol fixation, Giemsa staining, and microscopic examination. Approximately 2,000 erythrocytes were counted across randomly selected adjacent microscopic fields, and the percentage of parasitaemia was independently determined by two independent researchers. IC_50_ values were determined by nonlinear regression analysis of the dose–response curve (drug concentration versus percentage inhibition) using GraphPad Prism 8.

### 2.7 Statistical analysis

GraphPad Prism 8 was used to perform the statistical analysis.

#### 2.7.1 Gene expression analysis

To compare the expression of *PfHsp90β* within various asexual stages of the parasite, a Student’s *t*-test was performed (^*^p < 0.05, ^**^p < 0.01). The experiment was done with two biological replicates (*n* = 2), and mean ± SD was plotted.

#### 2.7.2 Yeast complementation assays

For analysing cell survival within three different strains we used one-way analysis of variance (ANOVA), in which we selected yeast strains harbouring an empty plasmid as the control group and compared the survival of *ScHsp82-* and *PfHsp90β-*harbouring strains with respect to the control using Dunnett’s multiple comparison test. The ^****^p-values < 0.0001 show highly significant differences. The cell survival for all strains was repeated at least three times (*n* = 3), and the mean ± SD was plotted.

#### 2.7.3 Drug sensitivity assays

For the inhibitor assay, we used one-way ANOVA, and we compared the survival of *PfHsp90α* and *PfHsp90β* strains with respect to the *ScHsp82-*harbouring strain (control) using Dunnett’s multiple comparison test (*p*-values > 0.05 are not significant [NS]; ^***^p-values < 0.001 are significant). The cell survival for each strain was repeated at least three times (*n* = 3), and mean ± SD was plotted.

#### 2.7.4 IC_50_ determination

Parasite growth inhibition assays were performed using two independent biological replicates. Dose-response curves were generated by plotting parasite growth inhibition against inhibitor concentration, and IC_50_ values were determined by nonlinear regression using a four-parameter logistic (4PL) model.

### 2.8 Sequence collection

Sequences for the Hsp90 family of homologous proteins were obtained from the National Center for Biotechnology Information (NCBI), UniProt, and PlasmoDB databases, and the dataset was narrowed to include major Hsp90 paralogues (i.e., cytosolic Hsp90α, putative Hsp90s, mitochondrial TRAP1, ER-resident GRP94, and bacterial HtpG) from species represented by all isoforms to provide a balanced sample size and to reduce bias when grouped together. A total of 69 different sequences were compiled into a single FASTA dataset (accession numbers and gene identifiers used for the construction of the trees are indicated in **Supplementary Table S2**).

### 2.9 Sequence alignment and tree construction

Homologous Hsp90 sequences were aligned with MAFFT (v7.526) using the accuracy-oriented L-INS-i strategy (--localpair --maxiterate 1,000), which incorporates local pairwise alignment information and iterative refinement to improve alignment quality across conserved regions. The resulting 1,593-column alignment was trimmed with trimAl in automated mode (−automated1), a heuristic that automatically selects between gap- and similarity-based column filtering optimised for maximum-likelihood phylogenetic reconstruction. Trimming removed 1,009 poorly aligned positions, leaving a final alignment of 584 amino-acid columns across 69 sequences, which was used for tree inference.

Maximum-likelihood phylogenies (unrooted and rooted) were reconstructed in IQ-TREE (v3.1.3). The best-fit substitution model was selected by ModelFinder (−m MFP) under the Bayesian information criterion, which identified Q.YEAST + I + R5—an empirical yeast-derived amino-acid exchange-rate matrix combined with a proportion of invariable sites (+I) and a five-category FreeRate model of among-site rate heterogeneity (+R5)—as optimal for the dataset. Branch support was estimated from 1,000 ultrafast bootstrap (UFBoot) replicates (−bb 1,000) together with 1,000 SH-aLRT replicates (−alrt 1,000), with the thread count set automatically (−nt AUTO). For the rooted analysis, *E. coli* HtpG was used as the outgroup. The best-scoring maximum-likelihood tree (the IQ-TREE.treefile) was visualised and annotated in iTOL.

### 2.10 Structural prediction

The amino acid sequence of the PfHsp90β was obtained from PlasmoDB and submitted for the prediction of its three-dimensional structure via the AlphaFold protein structure prediction pipeline. The resulting model was visualised using UCSF Chimera (v1.19) and annotated for structural domains according to the known common architecture of Hsp90 proteins, including the N-terminal ATPase domain, middle domain, and C-terminal domain. Regions lacking defined secondary structure were classified as disordered.

### 2.11 Structural comparison of ATP-binding sites

The crystal structures of the N-terminal domains of Hsp90 from multiple species were retrieved from the Protein Data Bank: PfHsp90β (PDB: 3IED), PfHsp90α (PDB: 3K60), human Hsp90α (PDB: 1BYQ), human Hsp90β (PDB: 6N8Y), and yeast Hsp82 (PDB: 1ZWH). Structural visualisation and comparative analysis were performed using PyMOL (v3.1.6.1) to identify the conserved ATP-binding area between the Hsp90 isoforms as referenced in the literature (Meyer et al., 1997).

### 2.12 Multiple sequence alignment

The sequences that correspond to the middle domain of Hsp90 were aligned using the MUSCLE algorithm in MEGA software. The alignment concentrated on determining the conservation of the motif (Meyer et al., 2003), particularly the residues defined to be important in ATP hydrolysis.

### 2.13 Protein structure preparation

The crystal structures of the N-terminal domains of Hsp90 isomers were obtained from the PDB; PfHsp90β (PDB ID: 3IED), PfHsp90α (PDB ID: 3K60), and explicit preparations were made for each protein structure by removing solvent molecules and heteroatoms, adding polar hydrogen molecules, and assigning the correct charge to perform docking. These preparations were performed in PyMOL 2.6 (LTS).

### 2.14 Ligand selection and preparation

A panel of 13 established Hsp90 inhibitors was curated through an extensive literature survey, each of which had previously been reported to inhibit Hsp90 activity; ATP was additionally included as the natural ligand and as an internal reference for the screen. Three-dimensional structures of all compounds were obtained from public chemical repositories (PubChem) or generated from their canonical SMILES where a deposited structure was unavailable, and imported into Open Babel. Each ligand was assigned the appropriate protonation state at physiological pH (~ 7.4), and explicit hydrogens were added, after which the geometry was refined by energy minimisation using the MMFF94 force field (steepest-descent followed by conjugate-gradient steps until convergence) to relieve steric clashes and yield a stable low-energy conformer. Gasteiger–Marsili partial charges were then assigned to each atom, nonpolar hydrogens were merged, and rotatable bonds were detected automatically before every ligand was converted to the PDBQT format required by AutoDock Vina and Smina.

### 2.15 Molecular docking

Molecular docking was carried out using AutoDock Vina (v1.2.7) and independently repeated in Smina to cross-check the predicted affinities. The prepared receptor and ligand structures were converted to the PDBQT format required by both engines, with the receptor treated as rigid and the rotatable bonds of each ligand allowed to flex. For each isoform, a search box was centred on the ATP-binding cleft of the N-terminal (Bergerat-fold) domain. For PfHsp90β (PDB: 3IED) the box was centred at *x* = − 29.057, *y* = − 23.712, and *z* = 11.496 Å with dimensions of 22 Å × 22 Å × 20 Å, and for PfHsp90α (PDB: 3K60) at *x* = 71.632, *y* = − 25.778, and *z* = 12.335 Å with dimensions of 20 Å× 20 Å× 20 Å; in each case, the box was large enough to enclose the entire nucleotide pocket whilst excluding the rest of the domain. Docking was performed with an exhaustiveness of eight, and up to 10 binding modes (num_modes = 10) were generated per ligand. For every compound, the top-ranked pose—the conformation with the lowest (most negative) predicted binding free energy—was taken as the representative pose, and its affinity (ΔG, kcal/mol) was recorded for comparison across ligands and isoforms.

Each ligand was docked against both proteins using identical grid definitions and settings in the two engines. Smina was run with its default AutoDock Vina scoring function, which combines two attractive steric Gaussian terms (gauss[*o* = 0, *w* = 0.5, *c* = 8], weight: − 0.035579; gauss[*o* = 3, *w* = 2, *c* = 8], weight: − 0.005156), a steric repulsion term (repulsion[*o* = 0, *c* = 8], weight: 0.840245), a hydrophobic term (hydrophobic[*g* = 0.5, *b* = 1.5, *c* = 8], weight: − 0.035069), a nondirectional hydrogen-bond term (non_dir_h_bond[*g* = − 0.7, *b* = 0, *c* = 8], weight: − 0.587439), and a rotatable-bond (torsional) penalty (num_tors_div, weight: 1.923). All remaining parameters in both Vina and Smina were kept at their standard default values. As both programmes used the same underlying scoring function, the Vina and Smina affinities could be compared directly and provided a consistency check on the predicted binding energies (**Table 1**).

**Table 1:** Binding affinity of Hsp90 inhibitors towards PfHsp90β.

Compound	Vina (kcal/mol)	Smina (kcal/mol)
NVP-AUY922	-8.6	-8.8
CCT018159	-8.3	-8.2
Ganetespib	-8.3	-8.4
MPC-3100	-8.1	-8.2
Radicicol	-7.7	-7.7
Debio 0932	-7.6	-7.5
XL888	-7.6	-7.7
Geldanamycin	-7.5	-7.5
Retaspimycin Hydrochloride	-7.2	-7.2
Tanespimycin / 17 AAG	-7.2	-7.2
CNF2024	-7.1	-7.1
PU-29F	-7.0	-7.2
PU-H71 (Zelavespib)	-6.9	-7.1

Before screening, the docking protocol was validated by redocking the native cocrystallised ligand into each receptor under the same conditions. The nonhydrolysable ATP analogue AMP-PNP was redocked into PfHsp90β (3IED) and ADP into PfHsp90α (3K60); the top-ranked poses reproduced the experimentally observed binding geometries with root-mean-square deviations of 1.7 and 1.9 Å, respectively. As both values fell below the customary 2 Å acceptance threshold, the protocol was judged reliable for the subsequent inhibitor screen.

### 2.16 Selection criteria and filtering

A curated panel of 13 established Hsp90 inhibitors was assembled for screening (**Table 1**). The compounds selected were well-characterised, ATP-competitive inhibitors of the Hsp90 N-terminal (Bergerat fold) pocket with documented anticancer activity. The panel spanned the principal pharmacophore classes, namely; benzoquinone ansamycins (geldanamycin, tanespimycin/17-AAG, retaspimycin), resorcinols (radicicol, NVP-AUY922, ganetespib, CCT018159), purine-scaffold inhibitors (PU-H71, PU-29F, BIIB021, MPC-3100), and additional synthetic inhibitors (Debio 0932, XL888). All compounds were first docked against PfHsp90β, using the docked affinity of the natural ligand ATP as the cut-off; only those binding more favourably than ATP were retained. Shortlisted compounds were then cross-docked against PfHsp90α under identical conditions, and selectivity was defined as preferential binding to PfHsp90β over PfHsp90α.

## 3 Results

### 3.1 Phylogenetic placement of putative PfHsp90 within the Hsp90 family

Phylogenetic analysis of representative homologues of Hsp90, including bacterial and diverse eukaryotic descendants, was performed in order to assess whether putative PfHsp90 is a canonical member of the Hsp90 chaperone family. The dataset used for this analysis included proteins annotated as cytosolic Hsp90, mitochondrial TRAP1, endoplasmic reticulum resident GRP94, bacterial HtpG, and putative Hsp90 present in a number of apicomplexan parasites, including several *Plasmodium* species. The broad taxonomic sampling of Hsp90 paralogues allowed for resolution of the major Hsp90 sister groups and placed putative PfHsp90 relative to well-established reference sequences. The unrooted maximum-likelihood tree resolved the sequences into five well-separated clades, including four major Hsp90 paralogues, namely, HtpG, TRAP1, GRP94, cytosolic Hsp90, and one clade of apicomplexan/protist putative Hsp90 (**Figure 1A**). Sequences of *Plasmodium* species formed a unique branch of the apicomplex/protist Hsp90 clade, and putative PfHsp90 clustered closely to Hsp90 homologues of *Plasmodium* species.

**Figure 1:**
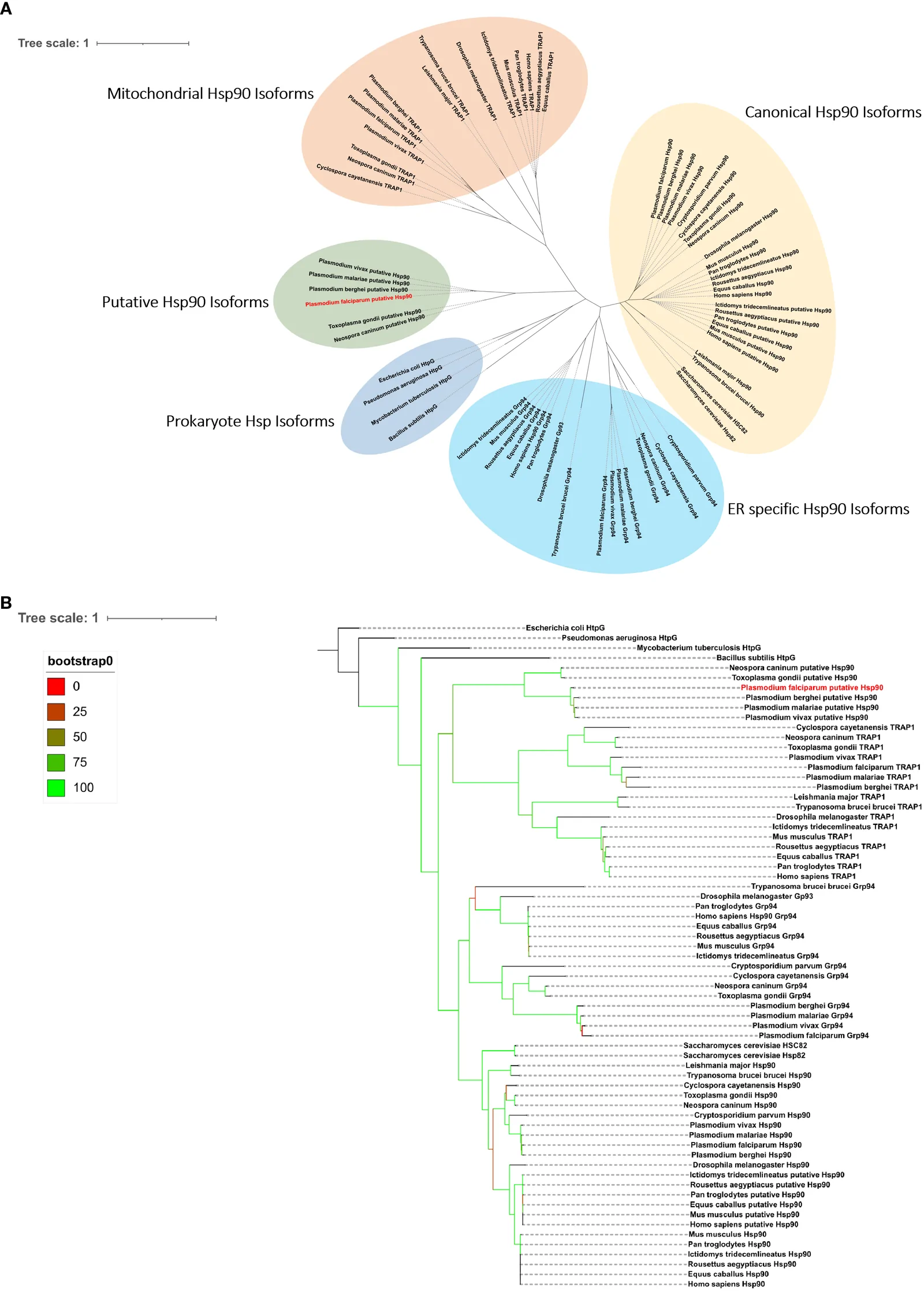
Phylogenetic placement of putative PfHsp90 within the Hsp90 family. **(A)** An unrooted phylogenetic tree was constructed using 69 Hsp90 protein sequences obtained from various databases. The dataset included representative sequences of cytosolic Hsp90a, putative Hsp90 isoforms, mitochondrial TRAP1, ER-resident GRP94, and bacterial HtpG across diverse taxa. The tree resolved into five distinct clades, as follows: bacterial HtpG (violet), mitochondrial TRAP1 (orange), ER-resident GRP94 (blue), cytosolic Hsp90a (yellow), and a distinct clade of putative PfHsp90 isoforms (green). Putative PfHsp90 (marked in red) clustered with other uncharacterised Hsp90s from *Plasmodium* and other apicomplexan homologues. In contrast, Hsp90 isoforms from metazoans clustered closer to their respective Hsp90a counterparts, indicating divergence between apicomplexan and nonapicomplexan sequences. **(B)** Using the same dataset, a rooted maximum likelihood tree was constructed with the outgroup as *Escherichia coli* HtpG. Similar to the unrooted tree, the overall tree topology supported the placement of the primary Hsp90 paralogues in separate clades. The rooted tree demonstrated how bacteria (HtpG) diverged first, with organelle-specific (TRAP1 and GRP94) paralogues separating from cytosolic Hsp90 paralogues. Putative PfHsp90 was represented in a separate apicomplexan-related branch. Branch colouration represented bootstrap values (0–100) based on 1,000 ultrafast bootstrap replicates and provided statistical evidence of the inferred tree topology, where the branch lengths represented amino acid substitutions per site.

The presence of highly similar homologues across multiple species suggested that this protein had been evolutionarily retained within the genus. Putative PfHsp90 was positioned within the inferred evolutionary relationships amongst homologous proteins from several *Plasmodium* species (**Figure 1B**). Moreover, it was distinct from all other (non-*Plasmodium*) protist homologues. The rooted tree further validated the topology and confirmed how quickly the bacterium diverged and how the two organelle-specific paralogous genes—mitochondrial TRAP1 and ER-localised GRP94—separated from cytosolic Hsp90 proteins. In both rooted and unrooted analyses, the putative PfHsp90 and the corresponding putative isoforms of other *Plasmodium* species formed a distinct clade that resolved separately from both the canonical cytosolic Hsp90s and the organelle-specific paralogues (mitochondrial TRAP1 and ER-localised GRP94). This placement indicated that the putative isoforms were not organelle-targeted paralogues. Given its separation from the organelle paralogue as well as the canonical Hsp90 clade, we refer to this isoform as PfHsp90β hereafter.

### 3.2 The ATP-binding domain is conserved in PfHsp90β

The domain architecture of PfHsp90β was modelled using AlphaFold (Jumper et al., 2021) to create a structural model of the protein (**Figure 2A**). The schematic representation of its domain organisation showed the presence of a typical three-domain configuration, characteristic of all Hsp90 family members (**Figure 2B**). The N-terminal domain (spanning from 99 to 349 amino acids) formed the canonical Bergerat fold containing an ATP-binding pocket. We compared the ATP-binding sites of PfHsp90β (PDB: 3IED) with those of PfHsp90α (PDB: 3K60), HsHsp90α (PDB: 1BYQ), HsHsp90β (PDB: 6N8Y), and yeast Hsp82 (PDB: 1ZWH) (**Figure 2C**). We observed that three conserved motifs, B1 (FLRELISNSSDATD; highlighted in red), B2 (DNGIGMT; yellow), and B3 (QFGQFGVGFYS; highlighted in green), forming the nucleotide-binding pocket and responsible for ATP binding and hydrolysis, remained conserved in the PfHsp90β isoform. The conservation of the ATP-binding cavity for PfHsp90β, as visualised as an orange mesh, indicated that PfHsp90β might have a functional ATP-dependent chaperone activity. Likewise, the ATP-binding cavities of the remaining isoforms were mapped within their respective N-terminal domains and visualised as coloured meshes: a blue mesh for PfHsp90α, a pink (magenta) mesh for HsHsp90α, an olive mesh for HsHsp90β, and a cyan mesh for yeast Hsp82 (ScHsp82). In each isoform, the cavity occupied an equivalent position within the Bergerat fold and remained bordered by the same three conserved motifs, B1 (red), B2 (yellow), and B3 (green). The comparable location, shape, and volume of this pocket in PfHsp90β relative to the catalytically active PfHsp90α, HsHsp90α, HsHsp90β, and ScHsp82 indicated that the nucleotide-binding cavity was structurally conserved across yeast, human, and *Plasmodium* isoforms, further supporting the conclusion that PfHsp90β retained a functional ATP-dependent chaperone activity.

**Figure 2:**
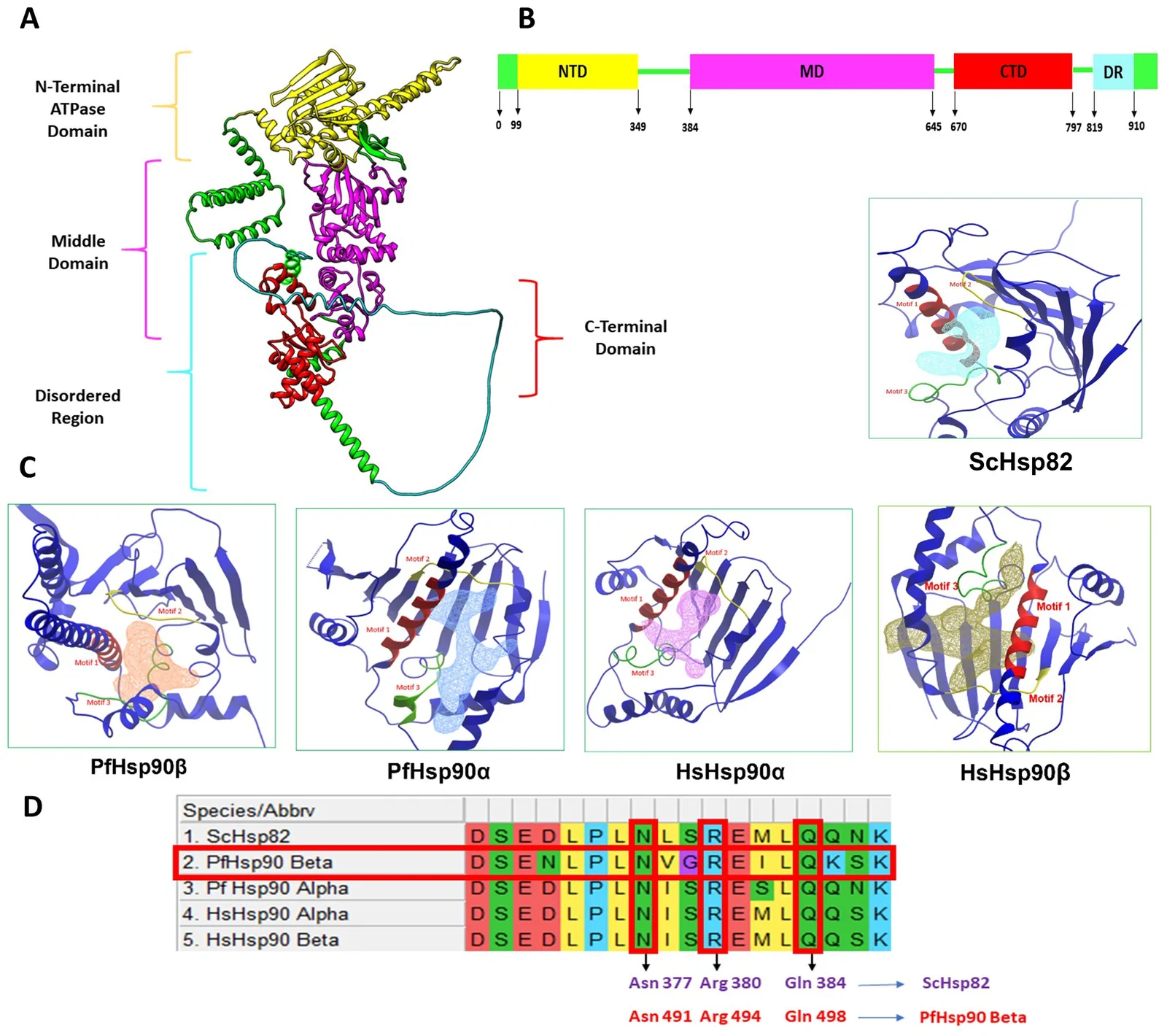
ATP-binding domain is conserved in PfHsp90b. **(A)** Schematic representation of domain organisation in PfHsp90b showing the N-terminal domain (NTD), middle domain (MD), C-terminal domain (CTD), and disordered region (DR) according to the AlphaFold prediction. **(B)** Visualisation of their three-dimensional organisation. **(C)** The ATP-binding site of PfHsp90b was compared with that of PfHsp90a, HsHsp90a, HsHsp90b, and ScHsp82, with three conserved motifs, B1 (FLRELISNSSDATD; red), B2 (DNGIGMT; yellow), and B3 (QFGQFGVGFYS; green) highlighted in the protein structure. **(D)** Multiple sequence alignment of the middle domain region of PfHsp90b with that of the other isoforms showed the presence of highly conserved Asn, Arg, and Gln residues implicated in ATP hydrolysis across species. Residue numbers of the conserved amino acids corresponding to yeast and PfHsp90b were indicated in purple and red, respectively.

The middle domain (including 384 to 645 amino acids) (**Figure 2B**) corresponded to the client-binding domain, which was also involved in the regulation of its ATPase activity. The multiple sequence alignment of the middle domain region revealed conservation of key motifs and amino acids as presented (**Figure 2D**). In yeast Hsp82, the motif [N(L/I/V)SREXLQ] corresponding to 377 to 384 amino acids had been implicated in ATP hydrolysis through its role in positioning catalytic residues. The three residues, Asn, Arg, and Gln, were shown to be the key interacting sites for the γ-phosphate of ATP (Meyer et al., 2003). We observed that these three key residues (N, R, and Q) were conserved amongst yeast, human, and *Plasmodium* isoforms. The high conservation of these residues across phylogenetically distant species supported the presence of an evolutionarily conserved functional core for ATP hydrolysis.

Although the catalytic pocket of PfHsp90β remained highly conserved, its uniqueness lay entirely in the noncatalytic positions that remained surrounding the ATPase pocket. Across these positions, the canonical cytosolic chaperones are remarkably alike: cytosolic PfHsp90α superimposes on human Hsp90 to ~ 0.2 Å across the active site and is approximately 65% identical overall, differing at only a handful of pocket-lining residues (Ser52→Ala38, Lys112→Arg98, Val186→Ile173, with a Met98→Met84 rotamer shift) that make its cavity modestly more constricted, basic, and hydrophobic (Corbett and Berger, 2010). The two human cytosolic isoforms are also very similar, sharing approximately 95% identity across the ATP pocket and differing at only two positions; thus, all four human isoforms are more than 85% identical in this region (Serwetnyk and Blagg, 2021). Against this backdrop of near-identical canonical isoforms, PfHsp90β stands out as the clear outlier.

The C-terminal domain of PfHsp90β corresponds to 670 to 797 amino acids (**Figure 2B**), which might be involved in dimerisation. The predicted structure lacked a clearly defined charged linker between the N-terminal and middle domains, which is typically seen in canonical cytosolic Hsp90s, and had an unexpectedly more compact transition between domains. In addition, there was an extended 91-amino-acid-long disordered region (DR) at the C-terminus (819 to 910 amino acids), which was thought to provide flexibility to the protein and might play a regulatory role during protein–protein interactions. The DR region provided unique characteristic features that were absent in classical cytoplasmic Hsp90. Additionally, it lacked the canonical MEEVD motif at the extreme C-terminus, which was involved in the binding of the TPR-domain-containing cochaperones in the cytosol. Collectively, these structural features suggested that PfHsp90β is a structurally unique member of the Hsp90 family and still maintains the essential core structure needed for its activity as a chaperone. Whilst deviations such as the absence of the charged linker and MEEVD motif distinguish it from canonical cytosolic isoforms, the preservation of the Bergerat fold and ATPase-associated residues supports its classification within the Hsp90 family.

### 3.3 *Plasmodium* Hsp90β has chaperone-like activity

To decipher the function of PfHsp90β, we first studied its expression within the parasite. We isolated RNA from synchronous ring, trophozoite, and schizont stage-specific parasites and performed semiquantitative RT-PCR to compare its stage-specific expression. We amplified 317 bp of *PfHsp90β* and 300 bp of Aspartate-rich protein (*ARP*) transcripts using gene-specific primers, with *ARP* acting as a loading control due to its constitutive expression at all the developmental stages of the parasite (Chalapareddy et al., 2014). We observed that *PfHsp90β* expression was highest at the trophozoite stage of the parasites (**Figure 3A**). We repeated this experiment with two independent biological replicates and observed similar results. The quantification of the gel images from two independent experiments revealed that *PfHsp90β* expression was nine- and threefold higher in the trophozoite (T) and schizont (S) stages of the parasites, respectively, compared to the ring stage (R) (**Figure 3B**), indicating its function in the T/S stages of the parasite. Previously, using yeast as a surrogate system, it was demonstrated that PfHsp90α could genetically complement yeast Hsp90 (Wider et al., 2009). In order to decipher whether PfHsp90β could fully complement yeast Hsp90, we used a plasmid shuffling assay. We used the P82a (*Δhsp82*, *Δhsc82*) yeast strain (Suhane et al., 2014), which was maintained by a single-copy plasmid (*TRP* marker) expressing *ScHsp82*. We tried to generate a strain that could be maintained by a single-copy PfHsp90β-expressing plasmid (*HIS* marker) by shuffling the original *TRP* plasmid; however, we could not generate the strain after several trials. This suggested that PfHsp90β could not fully complement yeast Hsp90 function. We anticipate that, due to the lack of the charged linker region as well as the MEEVD motif, PfHsp90β might not be able to function optimally within yeast utilising yeast cochaperones. We took another approach in which a well-characterised temperature-sensitive *iGD170hsp82* mutant strain (Nathan and Lindquist, 1995) was employed, in which endogenous copies of *Hsp82* and *Hsc82* were deleted, and the strain survived through a single mutant copy of *GD170hsp82*. At the permissive temperature (25 °C), the strain behaved as wild-type; however, if exposed to a nonpermissive temperature (37 °C) for a short duration, the strain exhibited growth defects due to nonfunctional *GD170hsp82*. We generated three isogenic strains in the aforementioned strain background, as depicted schematically (**Figure 3C**); one having an empty centromeric plasmid, and the others expressing the aforementioned vector containing either *ScHsp82* or *PfHsp90β*. The generation of the strains was confirmed by measuring the transcriptional expression of *ScHsp82* and *PfHsp90β* using gene-specific primers. All the strains with the *iGD170hsp82* background exhibited expression of *ScHsp82*; however, the strain harbouring an *ScHsp82* overexpressing plasmid displayed higher expression. The *PfHsp90β* expression was detected only in the strain harbouring its expression vector (**Figure 3D**). Each strain was grown in the exponential phase at 25 °C and divided into two batches; one of the batches was incubated at 37 °C for 4 h to allow functional inactivation of *GD170hsp82*, whilst the other batch continued to grow at 25 °C. We measured the per cent survival for each strain (**Figure 3E**) at 37 °C compared to that at 25 °C. The experiments for each strain were repeated three times, and our results showed that whilst the *iGD170hsp82* strain harbouring an empty plasmid showed only 11% growth, the same strain harbouring an *ScHsp82* overexpressing plasmid supported 69% growth (**Figure 3E**), which acted as a positive control in our assay system. The *PfHsp90β-*overexpressing strain, however, showed 37% survival, which was significantly higher than the empty vector-harbouring strain, but it was approximately 50% less than that of the positive control. The same experiment was conducted by incubating the strains at the restrictive temperature (37 °C) for 8 h. We observed a similar pattern that empty vector-harbouring strain failed to grow at 37°C and showed a reduction in cell survival (6%), whilst the strain harbouring *ScHsp82* showed 53% survival (**Figure 3F**). The *PfHsp90β-*expressing strain supported cell survival at a significantly higher ratio (27%) compared to the isogenic empty vector-harbouring strain. Together, our study establishes that PfHsp90β has Hsp90-compatible chaperone activity and can partially complement yeast Hsp82 chaperone function.

**Figure 3:**
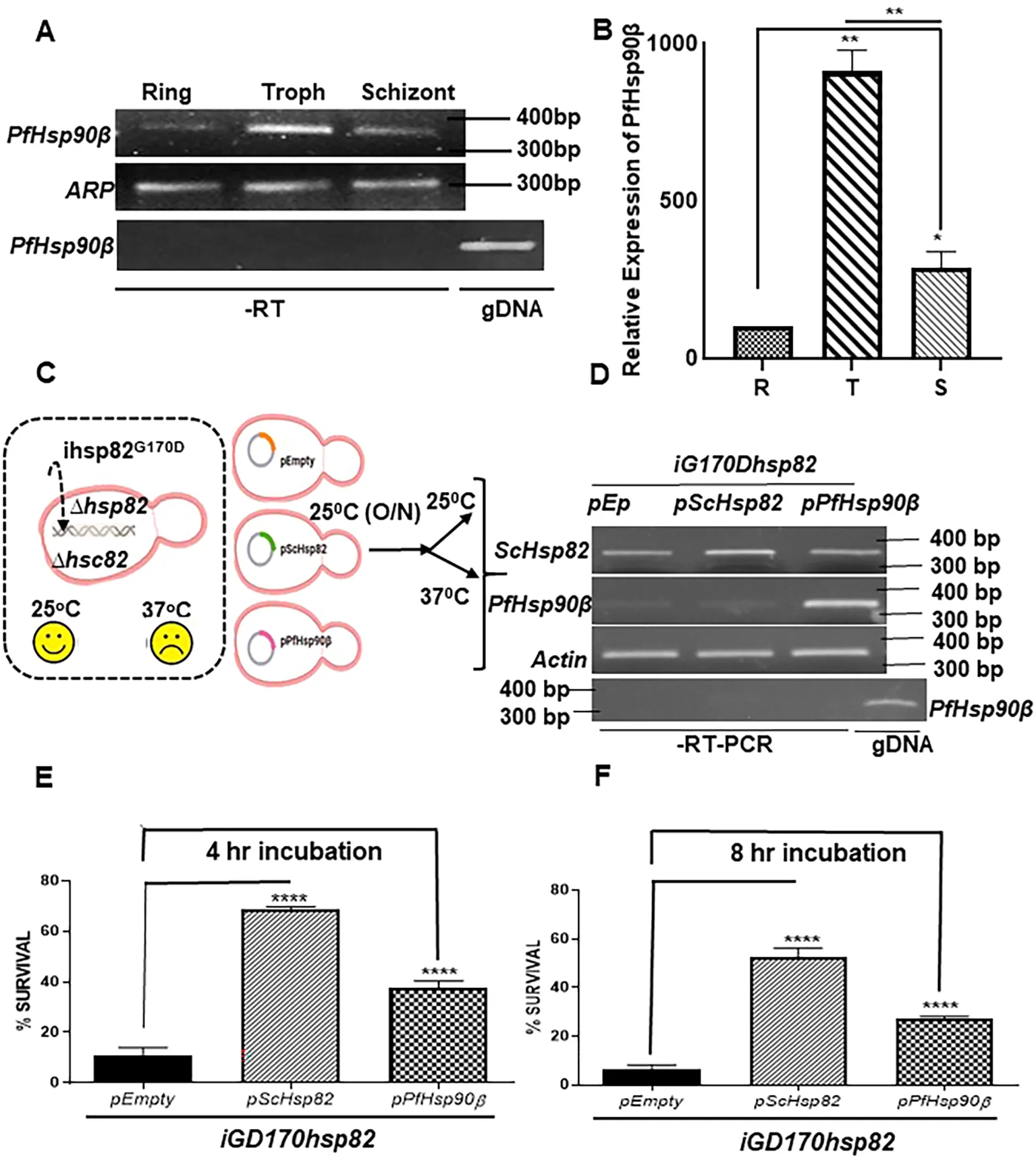
Plasmodium Hsp90b has chaperone-like activity. **(A)** RT-PCR analysis of RNA isolated from synchronised ring (R), trophozoite (T), and schizont (S) stages of the 3D7 parasite. PCR without reverse transcription (−RT) showed no amplification of PfHsp90b. Genomic DNA samples acted as a positive control. **(B)** The above experiment was repeated with two biological replicates, and quantification of PfHsp90b expression was performed using ImageJ; the mean value (± SD) of relative expression was plotted. The p-values were calculated using the two-tailed Student’s t-test. **p ≤ 0.01; *p ≤ 0.05. **(C)** Schematic representation of the yeast-based assay system. The temperature-sensitive iG170Dhsp82 strain (Dhsp82Dhsc82) harbours the mutated hsp82 integrated into the chromosomal locus, which behaves as wild-type when grown at a permissive temperature (25 °C) and fails to grow when shifted to a nonpermissive temperature (37 °C). Three isogenic strains were generated, each harbouring either ScHsp82 or PfHsp90b; the empty vector expressing strain acted as a negative control. **(D)** Transcriptional expression of PfHsp90b in the aforementioned strain was confirmed by amplifying cDNA with PfHsp90b-specific primers; PCR without reverse transcription (−RT) showed no amplification of PfHsp90b. **(E)** The iGD170hsp82 strain harbouring ScHsp82 or PfHsp90b was divided into two sets: one set was grown at 25°C, and the other was incubated at 37°C for 4 h or **(F)** 8 h, and per cent survival was determined. The ScHsp82-overexpressing strain could bypass the survival defects of empty vectorexpressing strains, acting as a positive control. The PfHsp90b-expressing strain displayed a significant increase in survival compared to the negative control for both 4 and 8 h of incubation at the restrictive temperature. Each experiment was repeated at least three independent times, and the mean (± SD) was plotted. The p-values were calculated using one-way ANOVA with Dunnett’s multiple-comparison test; ****p < 0.0001.

### 3.4 Identification of selective inhibitors against PfHsp90β through in silico ligand screening

To identify inhibitors of PfHsp90β, we used *in silico* approaches. Thirteen *bona fide* Hsp90 inhibitors were selected based on a literature survey. Each inhibitor was allowed to dock against the structures of PfHsp90β using two independent docking engines, AutoDock Vina and Smina, and their respective binding affinities were calculated (**Table 1**). The docking of the natural ligand ATP to PfHsp90β was presented (**Figure 4A**), and its binding affinity was calculated as − 7.5 kcal/mol. The nucleotide occupies the cavity (blue surface) with its adenine ring buried against the hydrophobic floor of the pocket and its phosphate tail directed towards the mouth of the site, where it remains solvent-exposed. This close shape complementarity between ATP and the cavity confirms both the location and the geometry of the active site and validates the docking protocol used for the subsequent screen. Using the ATP affinity as a threshold, the compounds that showed greater binding affinities than ATP were shortlisted as candidate inhibitors of PfHsp90β. The potent inhibitors of Hsp90α, namely: geldanamycin, 17-AAG, XL888, Debio 0932, and radicicol, showed comparable affinities towards PfHsp90β as those of ATP. However, NVP_AUY922, CCT018159, ganetespib, and MPC-3100 showed higher binding affinities. When comparing the binding affinities of the aforementioned inhibitors against PfHsp90α (**Table 2**), except for MPC_3100, all three showed higher affinities towards PfHsp90β than PfHsp90α. We chose one of the promising candidates, CCT018159, and investigated the structural basis of its binding. The active site of PfHsp90β was shown as cyan-coloured atoms in **Figure 4B**, with the residues lining the pocket (within 5 Å of the ligand) labelled in red; the docked ligand CCT018159 was drawn in purple and blue, and the figure was generated using PyMOL (v3.1.8). **Figure 4C** presents the corresponding two-dimensional interaction map of the same docked complex, in which every contact was colour-coded, so that the three-dimensional pose in **Figure 4B** and the full interaction inventory in **Figure 4C** could be read side by side. In the docked pose (**Figure 4B**), CCT018159 sat deep inside the ATP-binding cavity, threaded between the walls of the pocket so that its three ring systems—the ethyl-substituted resorcinol ring, the central methyl-pyrazole, and the fused benzodioxane—faced a different wall of the site. The most conspicuous features in **Figure 4B** were the yellow dashed lines marking the hydrogen bonds at roughly 2.9–3.3 Å. The structure of the CCT018159 molecule (**Figure 4C**), with a complete set of contacts, depicted how PfHsp90β engaged with different parts of the ligand. Reading the two panels together, the resorcinol ring acted as the principal anchor: its two hydroxyl groups formed a pair of conventional hydrogen bonds (green in **Figure 4C**) with Gly254 and Thr233, pinning that end of the molecule against the top of the pocket. The Gly254 contact was necessarily a main-chain (backbone) hydrogen bond, since glycine carried no side chain. At the opposite end of the ligand, the oxygen atom of the benzodioxan ring was linked to Asn224 by a hydrogen bond and to Glu220 by a weaker carbon–hydrogen bond (pale green in **Figure 4C**), together tethering this end of the molecule. Overlaid on this hydrogen-bond network, the positively charged side chain of Lys140 stacked over the face of one of the ligand’s aromatic rings to form a π–cation interaction (orange in **Figure 4C**), adding a strong, directional pull that a purely hydrophobic pocket would not provide. The remaining stabilisation was hydrophobic; the aromatic scaffold was packed against the nonpolar side chains of Ala137 and Met216 through π–alkyl and alkyl contacts (pink in **Figure 4C**), and was further boxed in by the hydrophobic wall of the pocket, including Phe257, Val255, Leu225, and the surrounding glycine-rich loop (Gly251, Gly256) seen in **Figure 4B**, which closed around the ligand and excluded solvent. Together, the combination of directional hydrogen bonding, an electrostatic π–cation anchor, and extensive hydrophobic burial suggested a favourable binding mode consistent with the observed docking score for PfHsp90β relative to both ATP and the classical Hsp90α inhibitors.

**Figure 4:**
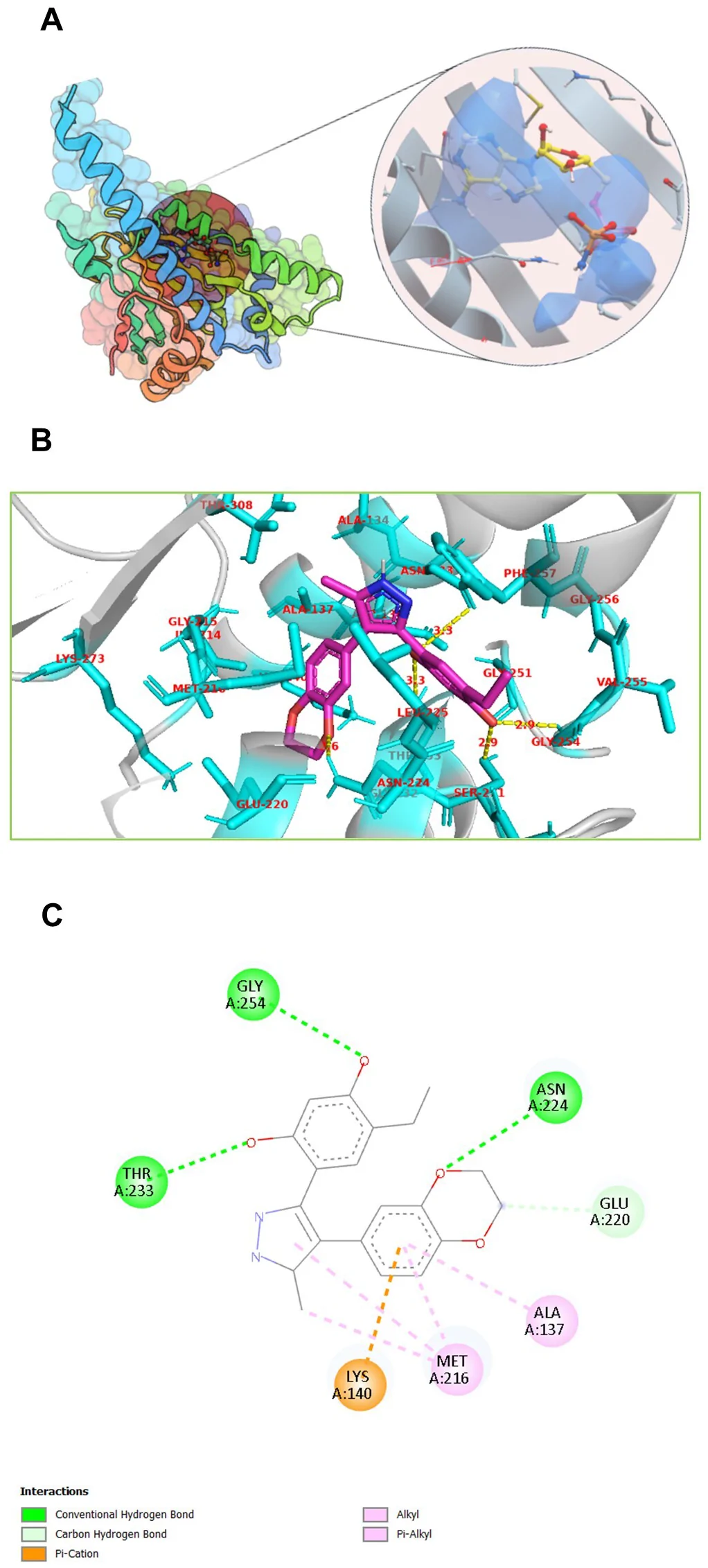
Identification of selective inhibitors against PfHsp90b through *in silico* ligand screening. Bona fide Hsp90 inhibitors were docked into the N-terminal ATP-binding domain of PfHsp90b (PDB ID: 3IED) using AutoDock Vina, and the binding of the top candidate, CCT018159, was analysed in detail. **(A)** Crystal structure of the N-terminal domain of PfHsp90b (PDB ID: 3IED) showing the ATP-binding (Bergerat fold) pocket. The full domain was shown as a cartoon, with the enclosed binding cavity rendered as a blue surface; ATP was docked within the cavity to define the boundaries and geometry of the active site. The inset showed a magnified view of the pocket with the bound nucleotide. **(B)** Molecular docking pose of CCT018159 in the active site of PfHsp90b. The active site was represented in cyan, and the residues lining the pocket (within 5 Å of the ligand) were labelled in red; the ligand was shown in purple/blue, and hydrogen bonds were drawn as yellow dashed lines. The image was rendered in PyMOL (v3.1.8). **(C)** Two-dimensional sketch of CCT018159 showcasing the protein–ligand interaction map of the same docked complex, summarising the full set of contacts stabilising CCT018159 in the pocket. The image was rendered in BIOVIA Discovery Studio Visualizer (v25.1.0.24284). Interactions were colour-coded by type: conventional hydrogen bonds (green), carbon–hydrogen bonds (pale green), p–cation (orange), and alkyl/p–alkyl hydrophobic contacts (pink), with the interacting residues indicated. **(B, C)** The three-dimensional binding pose and the corresponding interaction fingerprint of CCT018159 with PfHsp90b.

**Table 2:** Comparing binding affinities of inhibitors between PfHsp90α and PfHsp90β.

Hsp90 inhibitors	Vina (kcal/mol)	
	PfHsp90 β	PfHsp90 α
NVP_AUY922	-8.6	-7.8
CCT018159	-8.3	-7.6
Ganetespib	-8.3	-7.4
MPC_3100	-8.1	-8.2
Radicicol	-7.7	-7.6
ATP	-7.5	-7.5

### 3.5 Validation of PfHsp90β-specific inhibitors in yeast-based assay system

We wanted to determine whether any of the aforementioned compounds could cause functional inactivation of PfHsp90β. To that end, we tested whether PfHsp90β-dependent survival of the *iGD170hsp82* strain was affected at the restrictive temperature, in the presence of those inhibitors. We used the *Δpdr5-iG170Dhsp82* strain (SLY89) in which the Pleiotropic Drug-Resistance 5 (*PDR5*) protein, a component of a membrane-associated drug export pump, was deleted, ensuring optimal uptake of inhibitors. We generated three isogenic yeast strains in the above background, each expressing either ScHsp82 or PfHsp90α/PfHsp90β. Each strain was incubated at 37 °C for 4 h in the presence and absence of 2 µM radicicol, and per cent survival was measured. Whilst we observed that ScHsp82- and PfHsp90α-expressing strains showed 58% and 53% survival, respectively, the PfHsp90β-expressing strain did not show much inhibitory effect of radicicol and showed 88% survival under our assay conditions (**Figure 5A**). We used another potent inhibitor of PfHsp90α and ScHsp82, namely 17-AAG, and performed a similar assay. Our study showed that 20 µM 17-AAG treatment caused no survival defect in the PfHsp90β-harbouring strain, although that of ScHsp82 and PfHsp90α showed approximately 65% survival (**Figure 5B**). An earlier study also showed that yeast and PfHsp90α showed moderate inhibition by radicicol and 17-AAG in a yeast-based assay system (Wider et al., 2009), thus aligning well with our assay system. This correlated well with our *in silico* studies, in which these two compounds did not show stronger binding towards PfHsp90β. We used empirically determined concentrations of each of the four promising inhibitors, such as 2 µM NVP-AUY922 (**Figure 5C**), 50 µM CCT018159 (**Figure 5D**), 6 µM ganetespib (**Figure 5E**), and 20 µM MPC-3100 (**Figure 5F**), and performed the survival assay in a similar way with multiple repeats. Our study showed that only CCT018159 treatment caused a 40% decrease in survival of the PfHsp90β-expressing strain, whilst other strains were not significantly affected by those inhibitors at the concentrations being tested. Using different doses of CCT018159, we observed a dose-dependent decrease in survival of the PfHsp90β-harbouring strain (data not shown). Together, CCT018159 was shortlisted as the novel inhibitor that can selectively inhibit PfHsp90β.

**Figure 5:**
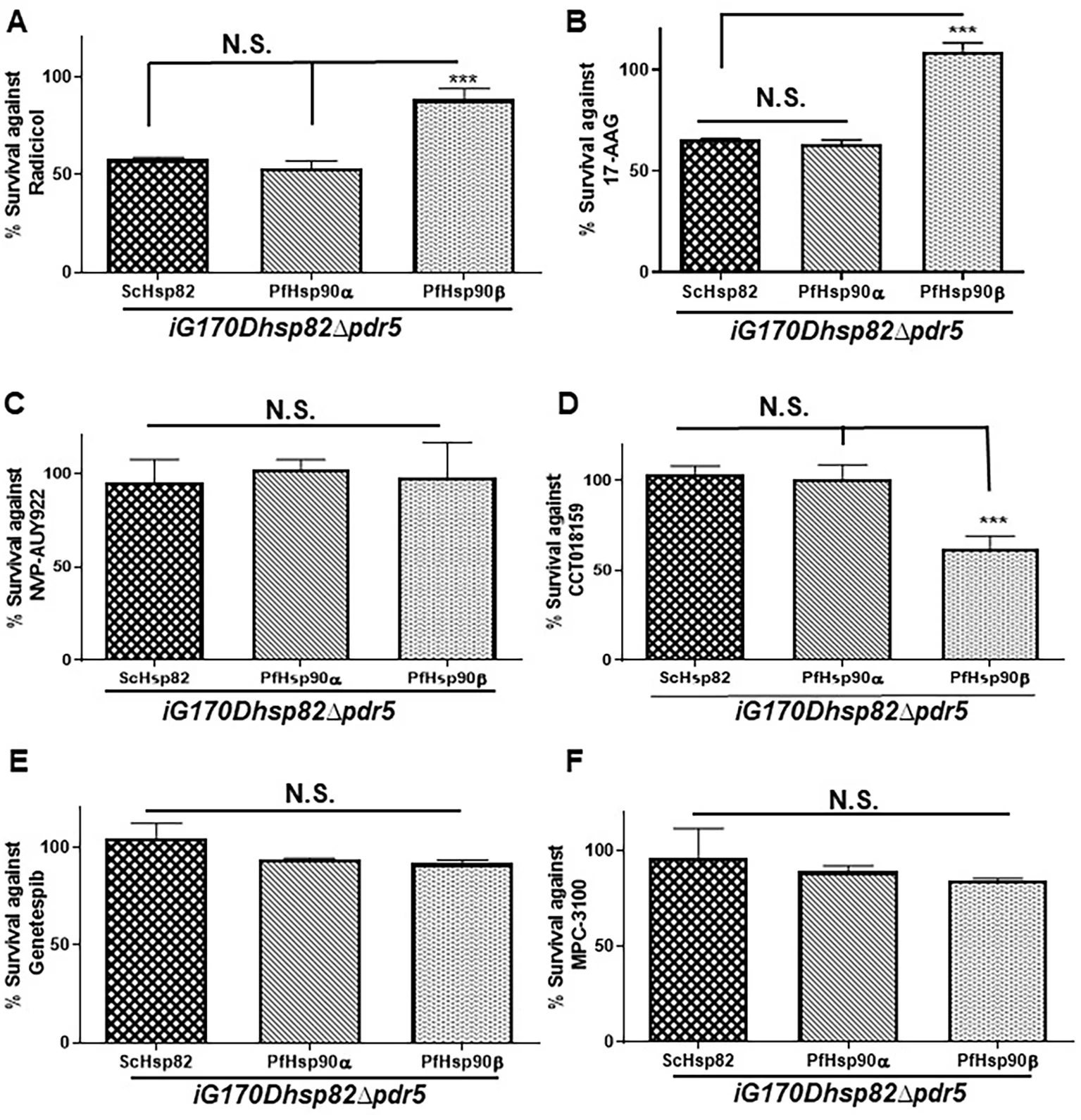
Validation of PfHsp90b-specific inhibitors in a yeast-based assay system. The *iGD170hsp82Dpdr5* strains harbouring various expression vectors (as shown on the x-axis) were grown at a restrictive temperature for 4 h in the presence and absence of **(A)** Radicicol, **(B)** 17-AAG, **(C)** NVP-AUY922, **(D)** CCT018159, **(E)** ganetespib, and **(F)** MPC3100, and per cent survival was plotted in each case. The PfHsp90b-expressing strain showed a significant decrease in survival only in CCT018159-treated cells. Each experiment was repeated three independent times, and the mean (± SD) was plotted. The p-values were calculated using one-way ANOVA with Dunnett’s multiple-comparison test, using ScHsp82-harbouring cells as a control. ***p < 0.001; NS, not significant.

### 3.6 Determination of IC_50_ of CCT018159 against drug-sensitive and drug-resistant strains of *Plasmodium falciparum*

To date, there is no report on the anti-*Plasmodium* activity of CCT018159. We exposed synchronous ring- and trophozoite stage-specific parasite cultures to CCT018159 and determined its anti-*Plasmodium* activity against drug-sensitive (3D7) and drug-resistant (Dd2) parasites. Typically, synchronous ring-stage-specific parasites were treated with different concentrations of CCT018159 for 48 h, and per cent inhibition was plotted against the concentration, and IC_50_ was determined. The IC_50_ values for CCT018159 were found to be 1.6 µM for 3D7 parasites (**Figure 6A**) and 2.8 µM for Dd2 parasites (**Figure 6B**). Furthermore, we used the trophozoite-stage-specific parasites and performed a similar assay. The per cent survival of the parasites was plotted after 48 h of incubation with the inhibitor. Our study showed that the IC_50_ values for 3D7 and Dd2 are 1.07 and 3 µM, respectively (**Figures 6C, D**). We conclude that both 3D7 and Dd2 parasites showed comparable IC_50_ values under R/T stage-specific conditions, although they differed slightly within themselves.

**Figure 6:**
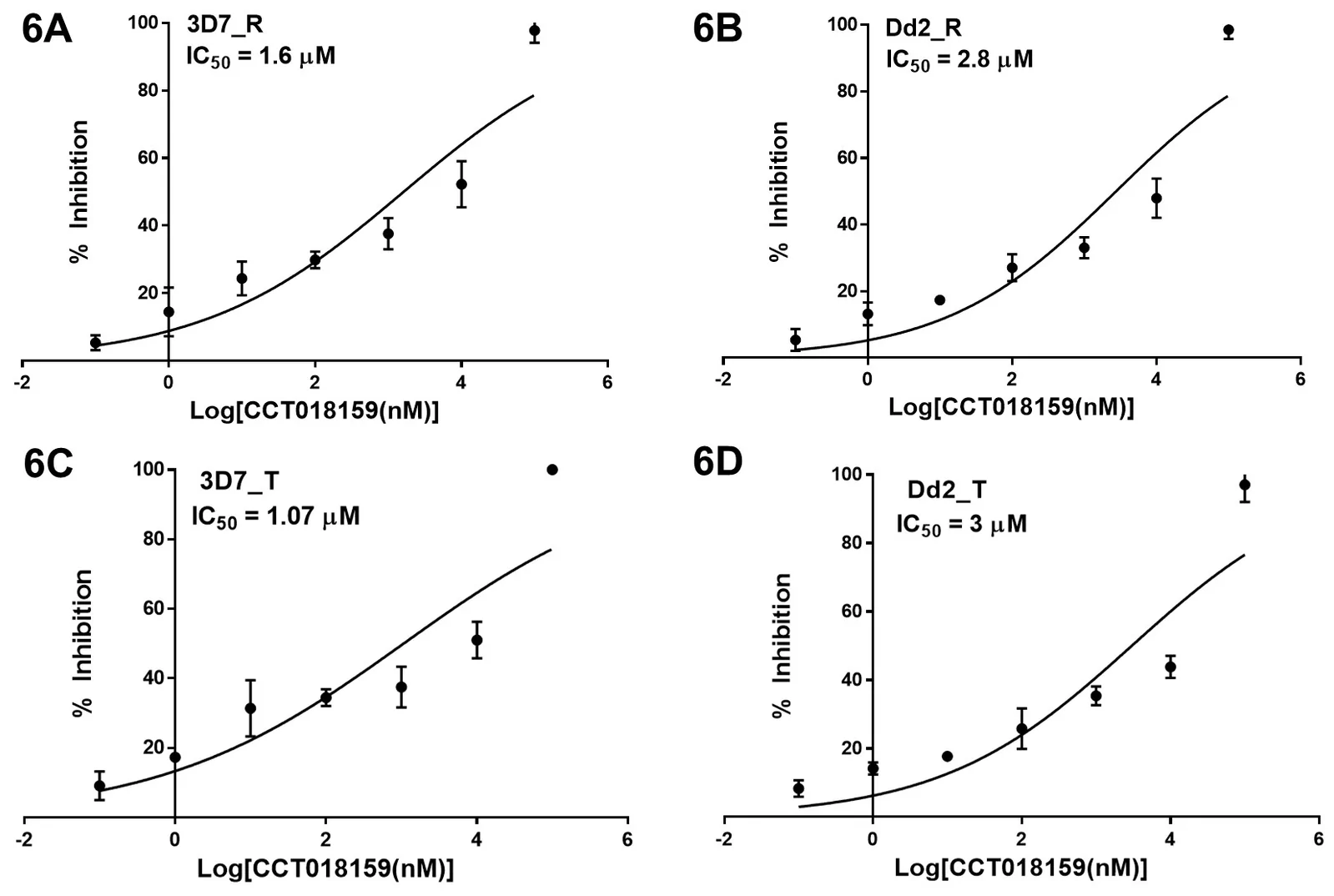
Determination of the IC_50_ of CCT018159 against drug-sensitive and drug-resistant strains of *Plasmodium falciparum*. **(A)** Synchronous ring-stage-specific 3D7 and **(B)** Dd2 were grown with various concentrations of CCT018159 for 48 h. **(C)** Trophozoite-stage-specific 3D7 and **(D)** Dd2 parasites were grown in the presence of different CCT018159 doses for 48 h. The parasitaemia at each dose of the inhibitor was measured using Giemsa staining, and per cent inhibition was plotted against the various doses of the inhibitor to obtain the IC_50_ values.

## 4 Discussions

The structure of the amino-terminal domain of PfHsp90β in a complex with an ADP analogue had been solved by the Structural Genomics Consortium at the University of Toronto (PDB: 3IED). In a phylogenetic study, PfHsp90β was resolved in a well-separated clade, distinct from both the canonical cytosolic clade and the human sequences, due to several unique structural features. Although the GHKL fold and catalytic core of PfHsp90β are preserved in this isoform, this degree of divergence implies a pocket lining that is substantially different from both PfHsp90α and the human isoforms. The differences extend beyond this to the overall domain architecture. PfHsp90β lacks the charged linker that normally connects the N-terminal and middle domains, instead adopting a compact N–M transition. It carries an unusually long C-terminal disordered region and lacks the terminal MEEVD motif that caps canonical cytosolic Hsp90s. These are not peripheral quirks: the charged linker is a principal modulator of N-domain conformation, transiently contacting the β-strands that build the pocket and thereby shaping its geometry (Lopez et al., 2021); hence, its absence implies an N-domain that has a different conformational landscape from that of PfHsp90α or human Hsp90. As the charged linker and the MEEVD motif are conserved features of human cytosolic Hsp90α/β, their loss marks PfHsp90β as architecturally distinct from the human isoforms as well as from PfHsp90α. Together, its phylogenetic isolation, its divergent pocket-lining sequence, and its atypical N-domain architecture identify PfHsp90β as a structurally unique member of the *Plasmodium* Hsp90 family.

The absence of MEEVD at the carboxyl-terminal end of PfHsp90β, as well as the lack of charged-linker residues, indicates a different cochaperone dependency of this chaperone. In eukaryotes, the TPR (tetratricopeptide repeat) domain-containing cochaperone Sti1/Hop bridges between Hsp70 and Hsp90 by interacting through their MEEVD motif and facilitates client transfer from Hsp70 to Hsp90 (Bhattacharya and Picard, 2021). The *in vivo* complex formation amongst *Plasmodium* Hsp70, Hsp90, and Hop has been established and was shown to be localised within the cytoplasm (Gitau et al., 2012). The bacterial orthologue of Hsp90, HtpG, however, lacks the MEEVD motif and has been shown to associate directly with DnaK (Hsp70 orthologue) along with DnaJ domain proteins during client transfer (Kravats et al., 2017). Future studies will reveal whether PfHsp90β follows similar mechanisms to those of HtpG. Moreover, the TPR domain-containing PfFKBP35 cochaperone interacts with PfHsp90 through the MEEVD motif (Alag et al., 2009), and their *in vivo* association has been detected within the parasite (Kumar et al., 2005). However, the physiological significance of this association in the malaria parasite has not been addressed so far. It remains to be deciphered whether PfHsp90β can facilitate client maturation in a PfFKBP35-independent manner.

It had been shown earlier that the charged linker deletion mutants of Hsp90 were conformationally restricted and, as a result, failed to bind P23 and were defective in Aha1-mediated ATPase stimulation (Hainzl et al., 2009). Unlike other eukaryotes, the apicomplexan parasites, including *Plasmodium*, harbour two paralogues of Aha1 and P23 cochaperones. It will be interesting to study whether PfHsp90β utilises those putative cochaperone(s) for its optimum function.

Poor amino-acid sequence homology of *Plasmodium* Hsp90β with that of the yeast orthologue and its unique cochaperone interaction partner might be responsible for its inability to fully complement ScHsp82 function during the plasmid shuffling experiment. However, by using a temperature-sensitive yeast strain, we could establish for the first time that PfHsp90β could partially sustain the growth of the yeast strain at the restrictive temperature.

In future, it will be necessary to characterise this chaperone by measuring its ATPase activity and its cochaperone dependencies by biochemical studies. We tried to express the full-length PfHsp90β protein using various bacterial host systems; unfortunately, we were not successful. We anticipate that the presence of the 100-amino-acid-long unstructured region at its amino-terminal domain might be the reason for its toxicity to the host, resulting in its lack of expression.

Due to the different configuration of the ATP-binding pocket in PfHsp90β, in our yeast-based assay, the PfHsp90β-harbouring strain showed resistance towards the *bona fide* Hsp90α inhibitors such as 17-AAG and radicicol. Furthermore, we observed that NVP-AUY922, ganetspib, and MPC-3100 showed no detectable inhibitory effects on any of the strains. We reason that deletion of only one transporter, *pdr5*, may not be enough to ensure entry for those three drugs. Multiple ABC transporter gene knockout strains (*Δsnq2*, *Δyor1*, *Δybt1*, *Δycf1*) might result in better sensitivities towards these drugs (Celaj et al., 2020), since transporter-mediated drug efflux is highly dependent on the physical shape and chemical properties of the drug. The inhibitory effect of CCT018159 on PfHsp90β but not on PfHsp90α correlates well with its decreased *in silico* binding affinities for PfHsp90α. CCT018159 was identified earlier as an inhibitor of human Hsp90β in a high-throughput screening assay (Sharp et al., 2007). Although it was reported that CCT018159 inhibited purified ScHsp82 with an IC_50_ of 6.6 µM, in our assay, we did not find any inhibitory effect in the *ScHsp82-*harbouring strain, even at 50 µM, because the inhibitor might require a large excess *in vivo* to compete with high concentrations of intracellular ATP for successful binding to ScHsp82. It is noteworthy that the selectivity of CCT018159 for PfHsp90β is primarily based on docking studies and inhibition studies in a yeast heterologous system. The *in silico* studies provide structural insights; however, they do not confirm that CCT018159 actually binds and inhibits PfHsp90β function. Future studies are required to establish that. It is important to determine the effect of this inhibitor on purified PfHsp90α as well as PfHsp90β to determine whether it shows selectivity for PfHsp90β.

The tightly regulated expression of this isoform at the metabolically active trophozoite stage suggests that the expression of its clients should cluster with PfHsp90β to maintain their cellular homeostasis. The effect of CCT018159 on the *Plasmodium* asexual developmental cycle indicates the importance of this isoform for parasite survival. Together, the unique sequence of PfHsp90β compared to its human orthologue and its negative mutant fitness score (MFS = − 3.033) qualify it as an antimalarial target. Our present study opens up further investigation into understanding the mode of action of this isoform of PfHsp90 and its substrate specificities.

## Data Availability

The original contributions presented in the study are included in the article/**Supplementary Material**. Further inquiries can be directed to the corresponding author.
