# Supplementary material for: Plasmodium falciparum putative Hsp90 functionally complements yeast Hsp90 and is inhibited in an isoform-specific manner
Source: Front Parasitol. 2026 Oct 12;5:1944050. doi: 10.3389/fpara.2026.1944050 (PMC13696465; doi:10.3389/fpara.2026.1944050)
Supplement: Supplementary file 1 [file Supplementaryfile1.zip › Supplementary Table S1.DOCX]

**Supplementary Table S1: Yeast Strains used in this study**

| **Strains** | **Genotype** |
| --- | --- |
| *P82a* | *MATa* *can1-100* *ade2-1* *his3-11,15* *leu2-3,112* *trp1-1* *ura3-1* *HSP82::LEU2* *HSC82::LEU2 CEN* pTGPD/P28 |
| *iG170Dhsp82* | *MATa* *can1-100* *ade2-1* *his3-11,15* *leu2-3,112* *trp1-1* *ura3-1* *HSP82::LEU2* *HSC82::LEU2* *HIS3::HSP82G170D* |
| *MBY1* | *MATa* *can1-100* *ade2-1* *his3-11,15* *leu2-3,112* *trp1-1* *ura3-1* *HSP82::LEU2* *HSC82::LEU2* *HIS3::HSP82G170D, pHCA* |
| *MBY2* | *MATa* *can1-100* *ade2-1* *his3-11,15* *leu2-3,112* *trp1-1* *ura3-1* *HSP82::LEU2* *HSC82::LEU2* *HIS3::HSP82G170D, pHCA-ScHsp82* |
| *MBY3* | *MATa* *can1-100* *ade2-1* *his3-11,15* *leu2-3,112* *trp1-1* *ura3-1* *HSP82::LEU2* *HSC82::LEU2* *HIS3::HSP82G170D, pHCA-PfHsp90β* |
| *DP533* | *Δhsc82::kanMx4Δhsp82:kanMx4/2µ-Hsc82-URA3 Δpdr::loxP-leu2-loxP, trp1-289, leu2-3, 112 his3-Δ200 URA3-52 ade2-101Δc lys2-801am* |
| *SLY89* | *Δhsc82::kanMx4Δhsp82:kanMx4/piHGpd-G170Dhsp82-HIS Δpdr::loxP-leu2-loxP, trp1-289, leu2-3, 112 his3-Δ200 URA3-52 ade2-101Δc lys2-801am* |
| *MBY4* | *Δhsc82::kanMx4Δhsp82:kanMx4/piHGpd-G170Dhsp82-HIS Δpdr::loxP-leu2-loxP, trp1-289, leu2-3, 112 his3-Δ200URA 3-52 ade2-101Δc lys2-801am, pTA-ScHsp82* |
| *MBY5* | *Δhsc82::kanMx4Δhsp82:kanMx4/piHGpd-G170Dhsp82-HIS Δpdr::loxP-leu2-loxP, trp1-289, leu2-3, 112 his3-Δ200URA 3-52 ade2-101Δc lys2-801am, pTA-PfHsp90α* |
| *MBY6* | *Δhsc82::kanMx4Δhsp82:kanMx4/piHGpd-G170Dhsp82-HIS Δpdr::loxP-leu2-loxP, trp1-289, leu2-3, 112 his3-Δ200URA 3-52 ade2-101Δc lys2-801am, pTA-PfHsp90β* |
