# Supplementary material for: Plasmodium falciparum putative Hsp90 functionally complements yeast Hsp90 and is inhibited in an isoform-specific manner
Source: Front Parasitol. 2026 Oct 12;5:1944050. doi: 10.3389/fpara.2026.1944050 (PMC13696465; doi:10.3389/fpara.2026.1944050)
Supplement: Supplementary file 1 [file Supplementaryfile1.zip › Supplementary Table S2.DOCX]

**Supplementary Table S2: Hsp90 family — accession list**

| **Accession ID** | **Name / Species** |
| --- | --- |
| **Canonical Hsp90 Isoforms** | |
| XP_034421027.1 | *Plasmodium_berghei* |
| XP_001613451.1 | *Plasmodium vivax* |
| XP_028859990.1 | *Plasmodium malariae* |
| XP_001348998.1 | *Plasmodium falciparum 3D7* |
| XP_002368278.1 | *Toxoplasma gondii* |
| XP_022589115.1 | *Cyclospora_cayetanensis* |
| XP_003881046.1 | *Neospora_caninum* |
| NP_005339.3 | *Homo_sapiens* |
| NP_001092042.1 | *Pan_troglodytes* |
| XP_077907057.1 | *Ictidomys_tridecemlineatus* |
| XP_016021504.2 | *Rousettus_aegyptiacus* |
| NP_001157427.1 | *Equus_caballus* |
| P02828 | *Drosophila_melanogaster* |
| NP_034610.1 | *Mus_musculus* |
| XP_626924.1 | *Cryptosporidium_parvum* |
| NP_015084.1 | *Saccharomyces_cerevisiae (Hsp82)* |
| NP_013911.1 | *Saccharomyces_cerevisiae (HSC82)* |
| XP_823307.1 | *Trypanosoma brucei brucei* |
| Q4Q4I6 | *Leishmania major* |
| **Putative Hsp90 Isoforms** | |
| XP_001348591.1 | *Plasmodium falciparum 3D7* |
| XP_034423231.1 | *Plasmodium_berghei* |
| XP_001615952.1 | *Plasmodium_vivax* |
| XP_028863409.1 | *Plasmodium_malariae* |
| XP_002364289.1 | *Toxoplasma gondii* |
| XP_003885054.1 | *Neospora_caninum* |
| XP_026193645.1 | *Cyclospor_cayetanensis* |
| NP_001258898.1 | *Homo sapiens putative* |
| NP_001075407.1 | *Equus_caballus* |
| XP_015984208.2 | *Rousettus_aegyptiacus* |
| XP_005318678.2 | *Ictidomys_tridecemlineatus* |
| NP_001267386.1 | *Pan_troglodytes* |
| P11499 | *Mus_musculus* |
| **Mitochondrial Hsp90 Isoforms** | |
| XP_001347859.1 | *Plasmodium_falciparum 3D7* |
| XP_034421649.1 | *Plasmodium_berghei* |
| XP_001615300.1 | *Plasmodium_vivax* |
| XP_028861599.1 | *Plasmodium_malariae* |
| XP_002370104.1 | *Toxoplasma_gondii* |
| XP_026194042.1 | *Cyclospora_cayetanensis* |
| XP_003879538.1 | *Neospora_caninum* |
| NP_057376.2 | *Homo_sapiens* |
| NP_001267429.1 | *Pan_troglodytes* |
| XP_005599065.3 | *Equus_caballus* |
| XP_016022037.1 | *Rousettus_aegyptiacus* |
| XP_005337798.1 | *Ictidomys_tridecemlineatus* |
| A1Z6L9 | *Drosophila_melanogaster* |
| Q9CQN1 | *Mus_musculus* |
| XP_828348.1 | *Trypanosoma_brucei_brucei* |
| XP_001686000.1 | *Leishmania major* |
| **ER specific Hsp90 Isoforms** | |
| XP_001350620.1 | *Plasmodium_falciparum* |
| XP_001617311.1 | *Plasmodium_vivax* |
| XP_034424197.1 | *Plasmodium berghei* |
| XP_028864459.1 | *Plasmodium_malariae* |
| XP_002366945.1 | *Toxoplasma_gondii* |
| XP_026192727.1 | *Cyclospora_cayetanensis* |
| XP_003882154.1 | *Neospora_caninum* |
| NP_003290.1 | *Homo_sapiens* |
| NP_001233416.1 | *Pan_troglodytes* |
| NP_001157345.1 | *Equus_caballus* |
| XP_015982486.2 | *Rousettus_aegyptiacus* |
| XP_005322404.2 | *Ictidomys_tridecemlineatus* |
| Q9VAY2 | *Drosophila_melangaster(Gp93)* |
| P08113 | *Mus_musculus* |
| XP_628530.1 | *Cryptosporidium_parvum* |
| XP_843950.1 | *Trypanosoma_brucei_brucei* |
| E9ADS8 | *Leishmania major* |
| **Bacterial Outgroup (Prokaryotic Hsp Isoforms)** | |
| P0A6Z3 | *Escherichia_coli_HtpG* |
| P46208 | *Bacillus_subtilis_HtpG* |
| P9WMJ7 | *Mycobacterium tuberculosis* |
| Q9I3C5 | *Pseudomonas_aeruginosa* |
